# Low Expression of DDX60 Gene Might Associate with the Radiosensitivity for Patients with Breast Cancer

**DOI:** 10.1155/2020/8309492

**Published:** 2020-07-20

**Authors:** Dongrun Xin, Jingfang Liu, Jincheng Gu, Yujie Ji, Jiawei Jin, Lu Sun, Qingliang Tai, Jianping Cao, Ye Tian, Hualong Qin, Zaixiang Tang

**Affiliations:** ^1^Department of Biostatistics, School of Public Health, Medical College of Soochow University, Suzhou 215123, China; ^2^Department of Gynaecology and Obstetrics, The First Affiliated Hospital of Soochow University, Suzhou 215123, China; ^3^State Key Laboratory of Radiation Medicine and Protection, Soochow University, Suzhou 215123, China; ^4^Department of Radiotherapy & Oncology, The Second Affiliated Hospital of Soochow University, Suzhou 215123, China; ^5^Department of Thoracic Surgery, The First Affiliated Hospital of Soochow University, Suzhou 215123, China; ^6^Jiangsu Key Laboratory of Preventive and Translational Medicine for Geriatric Diseases, Medical College of Soochow University, Suzhou 215123, China

## Abstract

DEXD/H box helicase 60 (DDX60) is a new type of DEAD-box RNA helicase, which is induced to express after virus infection. It might involve in antiviral immunity by promoting RIG-I-like receptor-mediated signal transduction. In addition, previous studies had shown that the expression of DDX60 is related to cancer, but there was still a lack of relevant research in breast cancer. In this study, we used the information of patients with breast cancer in the TCGA database for statistical analysis and found that the breast cancer patients with low expression of DDX60 exhibited radiosensitivity. Comparing the radiotherapy groups with the nonradiotherapy groups, for patients with low expression of DDX60, the adjusted hazard ratio (HR) values for radiotherapy were 0.244 (0.064–0.921) and 0.199 (0.062–0.646) in the training and validation datasets, with the *p* values 0.040 and 0.007, respectively. However, for patients with high expression of DDX60, the adjusted hazard ratio (HR) values were 3.582 (0.627–20.467) and 2.421 (0.460–12.773), with the *p* values 0.054 and 0.297, respectively. These results suggested that the expression of DDX60 might strongly associate with individualized radiosensitivity in patients with breast cancer.

## 1. Introduction

Breast cancer is one of the most common cancers in the world, accounting for a large proportion of cancer deaths in the world. The GLOBOCAN2018 showed that more than 2 million people were newly diagnosed with breast cancer in 2018, and nearly 627000 people died of breast cancer [1]. According to Chinese cancer statistics in 2015, breast cancer was the most common cancer among Chinese women, the number of breast cancer patients accounted for 15% of all female cancer patients [2]. For women aged 30 to 59, breast cancer was the most common diagnosed cancer. And it was also the leading cause of cancer death for women who are younger than 45 years old [2]. The treatment of breast cancer was mainly surgery, supplemented by radiotherapy and chemotherapy. To achieve better results, biotherapy could be joined. With the wide application of radiotherapy in clinical practice, researchers have paid more attention on how to make better use of radiotherapy to improve the life quality of breast cancer patients. Recently, there have been many studies on radiotherapy for breast cancer, and researchers have proposed some options about its improvement and regimens [3–5]. However, researchers did not have a unified view on the improvement of radiotherapy for breast cancer. The sensitivity of different individuals to radiotherapy was different. Therefore, it is hoped that patients with radiosensitivity can be predicted by testing potential biomarkers. Then, oncologists and surgeons can reduce adverse reactions and improve safety by adjusting the strategy of radiotherapy.

DEAD-box (DDX) protein, which is the largest family of RNA lyase, contains conserved amino acid Asp-Glu-Ala-Asp sequence and has 37 members in human beings. DDX protein could interact with rRNA, mRNA, and other RNAs to participate in DNA repair and proliferation, mRNA synthesis, RNA splicing, and modification. Simultaneously, it could also involve in translation initiation, ribosome and splice assembly, and cell cycle arrest and apoptosis [6]. As a transcription factor, the transcriptional product of DDX60 gene plays a significant role in human antiviral activities and interferon immunization. It has an enhanced influence on interferon response and antihantavirus effect [7]. Studies had shown that DDX60 was a new type of antiviral helicase, which could participate in viral ribonucleic acid degradation pathway and promote RIG-1-like receptor-mediated signal transduction. Furthermore, human DDX60 could function as a ligand-specific sentinel activated by RIG-1 and it was involved in RIG-1-mediated innate immune response in vivo [8, 9]. It was reported that DDX60 was defined as an outpost of the cytoplasmic antiviral response, which was counteracted by virus-mediated activation of the epidermal growth factor receptor [10]. In addition, some studies had shown that DDX60 gene was a new adverse subsite-specific biomarker for the occurrence and prognosis of oral squamous cell carcinoma (OSCC) [11]. From these studies, we speculated that DDX60 might be associated with other cancers. However, there was still a lack of research on the relationship between the expression levels of DDX60 gene and breast cancer.

We assumed that the expression levels of DDX60 might associate with radiosensitivity of patients. Radiosensitive patients could obtain better and safer survival status after radiotherapy. In order to verify our hypothesis, we analyzed the relationship between DDX60 and radiosensitivity of breast cancer based on TCGA, hoping to provide reference for the clinical treatment of breast cancer patients.

## 2. Data Sources and Methods

### 2.1. Data Sources

In this study, the data of gene expression and clinical information of breast cancer patients were derived from the TCGA database (http://cancergenome.nih.gov/). We obtained the DDX60 gene expression data and clinical information of 1097 patients through the TCGA-Assembler. First, we deleted the data of patients with no survival time and survival results. Then, we selected the patients with clear clinical records about radiotherapy and screened out the required 13 clinical factors from the existing data. Next, we combined the clinical data with the expression data of DDX60 at the level of mRNA. Finally, we summarized the comprehensive data and exactly got 700 patients to carry out this study.

### 2.2. Analytical Method

**Figure 1:**
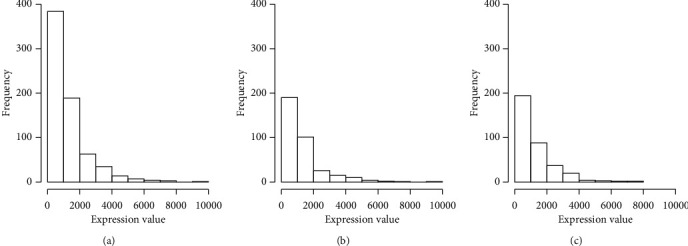
Expression distribution of DDX60 gene of patients with breast cancer. Expression distribution of DDX60 gene in all data. Expression distribution of DDX60 gene in training data. Expression distribution of DDX60 gene in validation data. (a) All patients. (b) Training patients. (c) Validation patients.

In this study, radiosensitivity was defined as the improved survival benefits of patients receiving radiotherapy. Then, the genes that could predict individual radiosensitivity were defined as radiosensitive genes. Their coding products could be used as potential biomarkers for radiosensitivity prediction. Since the expression distribution of DDX60 gene was skewness in training data (Figure 1), the median of expression was selected as the threshold of high and low expression. According to the previous study [11], we speculated that the expression of DDX60 might relate to the radiosensitivity of breast cancer patients. In order to verify that DDX60 in this study was a radiosensitive gene, we randomly divided the overall data into the training dataset and validation dataset. The training and validation datasets were equally analyzed as follows.

Then, univariate and multivariate Cox regression analyses were performed for patients with high and low expression. In this study, R software was used to make the survival curves in training and validation datasets. In addition, the logrank test and Cox regression analysis were used in our analysis. *p* < 0.05 was used as the criterion to verify statistical significance. The missing data were imputed by R software mice package.

## 3. Results

### 3.1. Correlation Analysis of DDX60 Expression and Clinical Indexes with Overall Survival

Using the Cox proportional hazard model, we analyzed the relationship between 13 clinical factors and the overall survival of breast cancer patients, and listed the HR (95% CI) and *p* values. Tables 1 and 2 show the analysis results of the training dataset and the validation dataset, respectively. The 13 clinical indicators including radiotherapy, age, history of other malignancies, histologic type, first surgical procedure, TNM stages, ER status, PR status, HER2 status, chemotherapy, and DDX60 expression level were included in multivariate analysis.

The results showed that there was no significant correlation between the expression levels of DDX60 and the overall survival of breast cancer patients. Radiotherapy is an effective treatment for breast cancer, but our results showed that radiotherapy did not significantly increase overall survival. Therefore, we inferred that not all breast cancer patients could acquire positive prognostic outcomes through radiotherapy. And those patients who obtained the improved survival benefits after receiving radiotherapy were the radiosensitive patient groups that we mentioned earlier. By looking for radiosensitive groups, we could determine which groups were more suitable for radiotherapy and then adjust the strategy of radiotherapy to significantly improve their survival rate.  Table 3 shows the chi-square test between other 12 clinical factors and the expression levels of DDX60, and the results showed that the expression level of DDX60 had no significant correlation with other 12 clinical factors.

### 3.2. Association Analysis of Radiotherapy and Expression Levels of DDX60

In order to further evaluate which groups were more suitable for radiotherapy, subgroup analysis was performed. The data in both training and validation datasets were classified as two groups with low and high expression levels of DDX60 gene. The two groups, respectively, adopted the Cox proportional hazard model.  Table 4 demonstrates the results of all groups. For the high-expression subgroup in training and validation datasets, radiotherapy showed higher risks than nonradiotherapy, which suggested that radiotherapy might reduce the survival rate of patients. Compared with nonradiotherapy, the adjusted HR for radiotherapy was 3.582 (0.627–20.467) and 2.421 (0.460–12.773) in training and validation datasets, with the *p* values 0.054 and 0.297, respectively. However, for the low-expression subgroup in training and validation datasets, compared with nonradiotherapy, the adjusted HR for radiotherapy was 0.244 (0.064–0.921) and 0.199 (0.062–0.646), with the *p* values 0.040 and 0.007, respectively, which indicated that radiotherapy could significantly improve the survival rate of patients with breast cancer. The data in both training and validation datasets were also mixed to analyze. Compared with nonradiotherapy, the adjusted HR for radiotherapy of the low-expression subgroup was 0.395 (0.186–0.840), while the high-expression subgroup was 1.687 (0.485–5.866). Results of mixed analysis and respective analysis were consistent.

**Figure 2:**
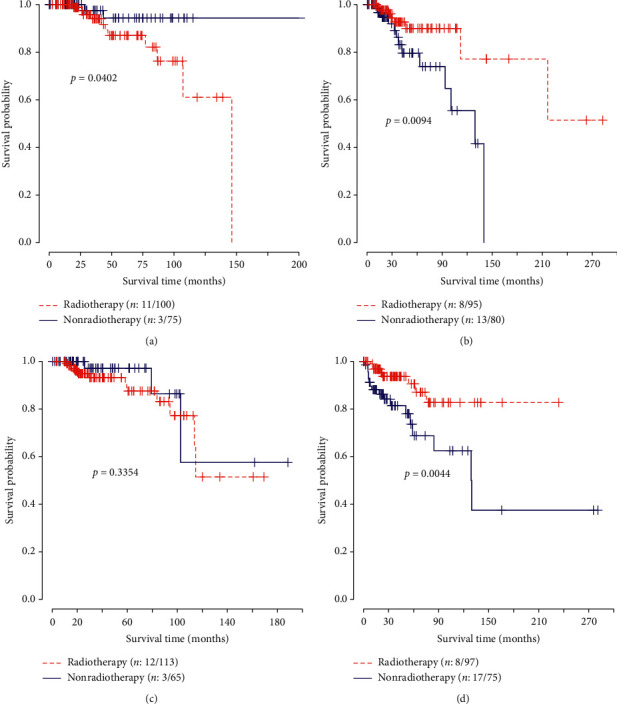
Survival curves under different expression levels of DDX60 in training and validation data. The logrank test was used to estimate the *p* values. The number before and after the slash referred to the number of deaths and sample size in subgroups, respectively. (a) High expressions in training. (b) Low expressions in training. (c) High expressions in validation. (d) Low expressions in validation.

Figure 2 illustrates the survival curves under different expression levels of DDX60 in training and validation data. As manifested in Figure 2, in the low-expression group, patients with radiotherapy showed better survival rate than those without radiotherapy in both training and validation data. The result possessed statistical significance. As for the high-expression group, patients with radiotherapy showed worse survival rate than those without radiotherapy in the training data, which meant that radiotherapy might reduce the survival rate. Patients with high expression levels of DDX60 were not suitable for radiotherapy in the training data. Nevertheless, in the validation data, there was not a significant association between radiotherapy and survival rate.

**Figure 3:**
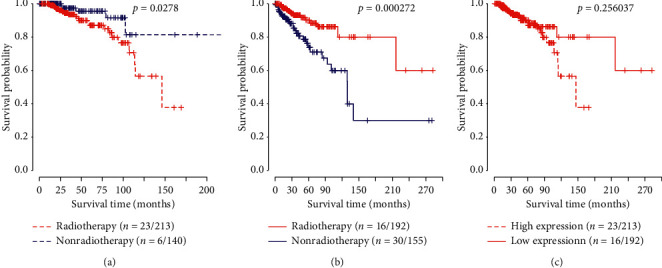
Survival curves under different expression levels of DDX60 for all patients. The logrank test was used to estimate the *p* values. The number before and after the slash referred to the number of deaths and sample size in subgroups, respectively. (a) High expression for all patients. (b) Low expression for all patients. (c) Patients with radiotherapy.

Figure 3 illustrates the survival curves of all patients under different expression levels of DDX60, which also proved the above results. From what have been discussed above, in the high-expression group, there was no significant improvement of overall survival between radiotherapy and nonradiotherapy groups, while in the low-expression group, the improvement among patients with radiotherapy was clear. The low expression levels of DDX60 gene could effectively predict the radiosensitivity of patients.

In addition, according to Tables 1 and 2, it was found that variables including age at diagnosis, TNM stages, and chemotherapy were statistically correlated with overall survival, indicating that effects of radiotherapy on survival probably needed further investigation under these subgroups. We performed subgroup analysis on age, TNM stages, and chemotherapy. The results are shown in Figures S1–S5. Similar conclusion could be drawn that patients with low expression level of DDX60 obtained relatively better survival benefits via radiotherapy than those with high gene expression level.

### 3.3. The Relationship between the Expression Levels of DDX60 and the New Tumor Event

**Figure 4:**
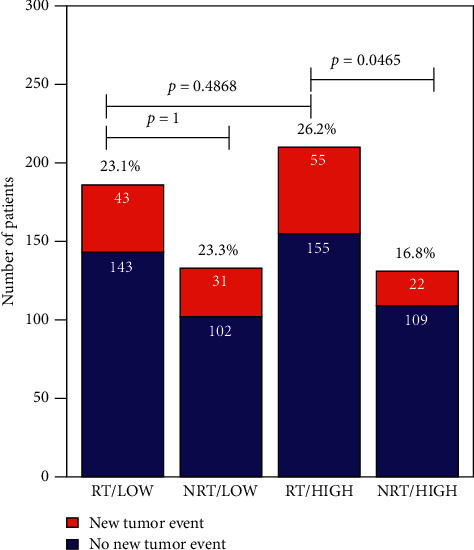
Associations between DDX60 expression levels and clinical assessment factors. The chi-square test was used for comparisons of rates of different groups. RT: radiotherapy; NRT: nonradiotherapy; HIGH: high expression of DDX60 gene; LOW: low expression of DDX60 gene.

Figure 4 demonstrates the relationship between the expression levels of DDX60 and the new tumor event. The new tumor event was made up of the in situ recurrence of breast cancer, distant metastasis, and other tumor-related events, which could be used as an indicator for prognosis. The results suggested that for the low-expression group, there was not a significant difference between new tumor event rates between radiotherapy and nonradiotherapy groups, while for the high-expression group, difference could be observed. For patients with high expression levels of DDX60, the new tumor event rate of the radiotherapy group was significantly higher than that of the nonradiotherapy group. It could be concluded that low-expression patients were suitable for radiotherapy, since radiotherapy would not increase their risks for new tumor event.

## 4. Discussion

Radiotherapy, as an indispensable treatment for breast cancer, could not only inhibit tumor growth but also decrease the mortality rate of patients. Under certain circumstances, radiotherapy could help some early-staged breast cancer patients and elderly patients with advanced breast cancer avoid operations [12]. However, radiotherapy might kill tumor cells and damage normal cells simultaneously. Previous research studies indicated that adjuvant radiotherapy might increase the cardiotoxicity of breast cancer patients [13]. Therefore, the focus of current research is the approach to conducting radiotherapy accurately and properly.

Our research discovered that radiotherapy was associated with the overall survival of patients by dividing patients into different groups according to their expression levels of DDX60. To some extent, the patients with low expression levels of DDX60 possessed radiosensitivity. In addition, the relationship between other clinical indicators and overall survival was also analyzed. The results found that overall survival was related to the age of the patients and TNM stages. The more accurate understanding of the association between the above clinical indicators and radiosensitivity of breast cancer could further promote the development of precise and individualized radiotherapy for breast cancer patients.

In our research, by adopting median division, we further divided the training data and validation data into low- and high-expression groups. Nine cutoff values based on different quantiles were chosen to evaluate the effects of radiotherapy on survival (Figure S6). We could find that when the cutoff was set to 5/10 quantile, the median, better, and significant radiotherapy effect was observed in the low-expression group; therefore, the median was selected as threshold of different expression levels.

Among all the patients who had received radiotherapy, based on the TCGA data, we compared indicators including radiation dose, type, and site between low- and high-expression groups, and no significant difference was observed, indicating that patients in these groups received the same type of radiation therapy (Table S1). The survival rate of the low-expression group was higher than that of the high-expression group. However, the *p* value was 0.256, which meant that the improvement of survival rate was not significant. It might be caused by the early censor and missing of data. The data of training and validation datasets showed that for the low-expression group of DDX60, the survival rate of the radiotherapy group was higher than that of the nonradiotherapy group, which meant that radiotherapy could increase the survival rate of low-expression patients. Meanwhile, radiotherapy might not increase or even decrease the survival rate of the high-expression patients, suggesting that radiotherapy was not suitable for patients with high expression levels of DDX60. It could be seen that the low expression levels of DDX60 might be used as an indicator for radiosensitivity of breast cancer.

In general, radiotherapy for breast cancer could be categorized into preoperative treatment, postoperative treatment, and palliative treatment. Among them, preoperative treatment could recognize the location of tumor, predict a series of biomarkers, and classify the risks for adjuvant treatment to avoid the delay of the local treatment [14]. In addition, the operation could remove radiated tissues after preoperative radiotherapy, allowing potential reradiation under the rescue circumstance. However, preoperative radiotherapy also has some risks, and the risks are associated with the sequences of treatment for patients. If the sequence is preoperative radiotherapy ⟶ neoadjuvant chemotherapy (NACT) ⟶ operation, risks of complications of delayed surgery after operation will increase [15]. Postoperative treatment is also vital, which can not only significantly decrease the recurrence rate and mortality rate of breast cancer but also improve the life quality of breast cancer patients [16–18]. Related studies showed that for the total resection of lymph node negative diseases with limited size and whole-body risks, conducting partial breast irradiation after breast-conserving surgery could shorten the stages [19, 20] and might have better beauty effects [21]. However, postoperative radiotherapy for breast cancer still has certain disadvantages. The common disadvantage is the radiopulmonary lesion of varying degrees, which is usually influenced by irradiation dose [22]. Besides, radiation might damage the capillary system and lead to fibrosis and failure of wound recovery [23]. Compared with preoperative treatment and postoperative treatment, palliative treatment is usually applied for patients whose cancer cannot be radically cured by radiotherapy and other treatment methods. Palliative treatment can reduce the nidus, relieve pain, and extend life so as to improve the life quality of patients. For example, previous research showed that palliative radiotherapy of retrobulbar metastases of breast cancer could reduce acute clinical symptoms [24]. In addition, palliative radiotherapy also played an important role in treating breast cancer with brain metastases and leptomeningeal carcinomatosis [25].

At present, although the relationship between DDX60 and radiotherapy for breast cancer remains unclear, the associations between DDX60 and antiviral immunity, colorectal cancer, and oral squamous cell carcinoma have been proved. Research showed that the helicase domain of purified DDX60 could bind viral RNA to DNA, to serve the purpose of antiviral immunity through promoting RIG-I-like receptor-mediated signaling [8]. It should be noted that RIG-I-like receptors are not only associated with antiviral immunity but also have an influence on inhibiting breast cancer [26], which implies the relationship between DDX60 and breast cancer to a certain extent. In addition, as a member of the DEAD-box protein family, DDX60 acts as a helicase, playing an important role in cellular life activities such as transcription, translation, and apoptosis. DDX60 also takes part in mRNA synthesis, RNA splicing and modification, DNA repairing and proliferation, etc. [27–33]. All these important activities, which are mentioned above, are associated with the occurrence and development of cancer in some degree. Related studies manifested that DDX60 showed high expression levels among OSCC patients. Tongue squamous cell carcinoma (TSCC) or OSCC patients with high expression levels of DDX60, particularly those with moderately or poorly differentiated tumors, showed a poor disease-free survival (DFS) [11]. Hence, the expression levels of DDX60 have certain relations with the occurrence, development, and prognosis of cancer.

In this study, we found that the low DDX60 expression group possessed radiosensitivity. Based on the mechanism of DDX60 interfering with breast cancer and other cancers, it was initially suspected that DDX60 might further influence resistance of tumor cells to radiation by influencing RNA synthesis, DNA repairing, and proliferation [34], which made patients with different DDX60 expression levels exhibit different radiosensitivity. Nevertheless, the specific mechanism remains to be discovered.

Herein, internal verification strategy was adopted, making up for the deficiency of small sample size to an extent. Additionally, under the circumstance that there were few studies concerning the relationship between DDX60 and radiosensitivity of breast cancer, our research has made some progress in the field of the relationship between gene and radiosensitivity, which might promote the individualized development of radiotherapy for breast cancer. However, the specific mechanism of how DDX60 interacts with radiosensitivity remains to be discovered. Meanwhile, a series of related external validation experiments need to be conducted, such as laboratory experiments and clinical experiments. Although our study had some limitations, the study still could offer some new directions for future research on both DDX60 and radiosensitivity of breast cancer.

## Figures and Tables

**Table 1:** Associations of clinical indicators and DDX60 expression levels with total survival in training data.

		Univariate analysis		Multivariate analysis	
		HR (95% CI)	*p* values	HR (95% CI)	*p* values
Radiotherapy					
Yes	195 (55.71%)	0.729 (0.372–1.426)	0.356	0.663 (0.280–1.571)	0.351
No	155 (44.29%)	1.000		1.000	
Age					
≥60	166 (47.43%)	2.424 (1.227–4.789)	0.011	2.404 (1.076–5.372)	0.033
<60	179 (51.14%)	1.000		1.000	
NA	5 (1.43%)				
History of other malignancies					
Yes	14 (4.00%)	2.768 (0.655–11.700)	0.166	1.334 (0.255–6.966)	0.732
No	336 (96.00%)	1.000		1.000	
Histologic type					
IDC	232 (66.29%)	0.830 (0.379–1.817)	0.641	1.514 (0.569–4.033)	0.406
MBC	28 (8.00%)	0.703 (0.185–2.671)	0.604	1.971 (0.469–8.291)	0.355
ILC	83 (23.71%)	1.000		1.000	
NA	7 (2.00%)				
First surgical procedure					
Lumpectomy	83 (23.71%)	0.387 (0.071–2.113)	0.273	0.430 (0.069–2.666)	0.364
Modified radical mastectomy	120 (34.29%)	2.442 (0.806–7.401)	0.114	1.740 (0.452–6.697)	0.421
Others	64 (18.29%)	1.565 (0.473–5.180)	0.463	1.581 (0.425–5.887)	0.495
A simple mastectomy	63 (18.00%)	1.000			
NA	20 (5.71%)				
T stage					
T3/T4	56 (16.00%)	2.980 (1.448–6.133)	0.003	1.328 (0.469–3.760)	0.594
T1/T2	293 (83.71%)	1.000		1.000	
NA	1 (0.29%)				
N stage					
N2/N3	61 (17.43%)	4.091 (1.996–8.384)	<0.001	3.525 (1.134–10.956)	0.030
N0/N1	286 (81.71%)	1.000		1.000	
NA	3 (0.86%)				
M stage					
M1	8 (2.29%)	4.233 (1.716–10.440)	0.002	1.741 (0.401–7.557)	0.459
M0	300 (85.71%)	1.000		1.000	
NA	42 (12.00%)				
ER status by IHC					
Positive	271 (77.43%)	2.996 (1.018–8.814)	0.046	1.870 (0.372–9.417)	0.448
Negative	64 (18.29%)	1.000		1.000	
NA	15 (4.29%)				
PR status by IHC					
Positive	234 (66.89%)	2.029 (0.863–4.772)	0.105	0.925 (0.260–3.290)	0.905
Negative	101 (28.86%)	1.000		1.000	
NA	15 (4.29%)				
HER2 status by IHC					
Positive	72 (20.57%)	0.791 (0.296–2.118)	0.641	0.856 (0.217–3.378)	0.826
Overexpression	51 (14.57%)	1.043 (0.335–3.244)	0.942	0.746 (0.151–3.682)	0.724
Negative	174 (49.71%)	1.000		1.000	
NA	53 (15.14%)				
Chemotherapy					
Yes	295 (84.29%)	0.237 (0.100–0.565)	0.001	0.214 (0.073–0.623)	0.005
No	55 (15.71%)	1.000		1.000	
DDX60 expression					
High	175 (50%)	0.7003 (0.353–1.391)	0.309	0.816 (0.379–1.757)	0.604
Low	175 (50%)	1.000		1.000	

*Note.* NA: data are not available; HR: hazard ratio; CI: confidence interval; IDC: invasive ductal carcinoma; MBC: special carcinomas, medullary carcinomas, mucinous carcinomas, mixed carcinomas, and others; ILC: invasive lobular carcinoma; IHC: immunohistochemistry.

**Table 2:** Associations of clinical indicators and DDX60 expression levels with total survival in validation data.

		Univariate analysis		Multivariate analysis	
		HR (95% CI)	*p* values	HR (95% CI)	*p* values
Radiotherapy					
Yes	210 (60.00%)	0.596 (0.320–1.108)	0.102	0.515 (0.221–1.200)	0.126
No	140 (40.00%)	1.000		1.000	
Age					
≥60	170 (48.57%)	2.296 (1.207–4.369)	0.011	2.593 (1.132–5.940)	0.025
<60	180 (51.43%)	1.000		1.000	
History of other malignancies					
Yes	22 (6.29%)	2.159 (0.760–6.131)	0.149	2.724 (0.767–9.676)	0.121
No	327 (93.43%)	1.000		1.000	
NA	1 (0.29%)				
Histologic type					
IDC	229 (65.43%)	0.861 (0.397–1.866)	0.704	1.948 (0.732–5.184)	0.182
MBC	33 (9.43%)	1.509 (0.561–4.061)	0.416	3.344 (0.948–11.794)	0.061
ILC	82 (23.43%)	1.000		1.000	
NA	6 (1.71%)				
First surgical procedure					
Lumpectomy	82 (23.43%)	1.570 (0.607–4.059)	0.352	1.392 (0.429–4.514)	0.582
Modified radical mastectomy	112 (32.00%)	1.159 (0.447–3.008)	0.761	1.070 (0.329–3.484)	0.911
Others	56 (16.00%)	0.498 (0.154–1.607)	0.243	0.413 (0.094–1.821)	0.248
A simple mastectomy	76 (21.71%)	1.000			
NA	24 (6.86%)				
T stage					
T3/T4	57 (16.29%)	1.429 (0.694–2.942)	0.333	1.402 (0.555–3.543)	0.475
T1/T2	293 (83.71%)	1.000		1.000	
N stage					
N2/N3	66 (18.86%)	2.112 (0.982–4.539)	0.056	3.440 (1.066–11.099)	0.052
N0/N1	277 (79.14%)	1.000		1.000	
NA	3 (2.00%)				
M stage					
M1	3 (0.86%)	14.508 (3.343–62.960)	<0.001	2.975 (0.685–12.920)	0.151
M0	297 (84.86%)	1.000		1.000	
NA	50 (14.29%)				
ER status by IHC					
Positive	266 (76.00%)	0.726 (0.316–1.667)	0.450	1.193 (0.375–3.793)	0.765
Negative	61 (17.43%)	1.000		1.000	
NA	23 (6.57%)				
PR status by IHC					
Positive	233 (66.57%)	0.6274 (0.321–1.227)	0.173	0.424 (0.146–1.232)	0.120
Negative	91 (26.00%)	1.000		1.000	
NA	26 (7.43%)				
HER2 status by IHC					
Positive	63 (18.00%)	1.205 (0.468–3.107)	0.699	1.354 (0.402–4.561)	0.632
Overexpression	40 (11.43%)	1.281 (0.370–4.440)	0.696	0.934 (0.143–6.090)	0.944
Negative	192 (54.96%)	1.000		1.000	
NA	55 (15.71%)				
Chemotherapy					
Yes	302 (86.29%)	0.678 (0.265–1.738)	0.419	0.517 (0.139–1.579)	0.247
No	47 (13.43%)	1.000		1.000	
NA	1 (0.29%)				
DDX60 expression					
High	178 (50.86%)	0.590 (0.310–1.123)	0.108	0.527 (0.251–1.105)	0.090
Low	172 (49.14%)	1.000		1.000	

*Note.* Abbreviations are the same as in Table 1.

**Table 3:** Relationship between expression levels of DDX60 and clinical indicators.

	Training data (*n* = 350)				Validation data (*n* = 350)			
	High	Low	*X* ^2^	*p* values	High	Low	*X* ^2^	*p* values
Radiotherapy			0.185	0.667			1.548	0.214
Yes	100	95			113	97		
No	75	80			65	75		
Age			0.561	0.454			0.175	0.675
≥60	79	87			84	86		
<60	96	88			94	86		
History of other malignancies			0.000	1.000			0.084	0.772
Yes	7	7			10	12		
No	168	168			167	160		
Histologic type			1.467	0.480			2.829	0.243
IDC	122	110			116	113		
MBC	12	16			21	12		
ILC	39	44			38	44		
First surgical procedure			0.701	0.873			1.211	0.750
Lumpectomy	40	43			40	42		
Modified radical								
Mastectomy	58	62			59	53		
Others	33	31			25	31		
A simple mastectomy	34	29			40	36		
T stage			1.091	0.296			0.186	0.666
T3/T4	24	32			27	30		
T1/T2	151	142			151	142		
N stage			0.675	0.411			0.156	0.693
N2/N3	34	27			36	30		
N0/N1	140	146			141	136		
M stage			0.070	0.791			0.002	0.963
M1	5	3			1	2		
M0	154	146			153	144		
ER status by IHC			0.197	0.657			0.867	0.352
Positive	133	138			138	128		
Negative	34	30			27	34		
PR status by IHC			0.041	0.840			3.496	0.062
Positive	118	116			126	107		
Negative	49	52			38	53		
HER2 status by IHC			2.748	0.253			2.706	0.259
Positive	42	30			38	25		
Overexpression	24	27			20	20		
Negative	82	92			93	99		
Chemotherapy			4.228	0.040			0.537	0.464
Yes	155	140			156	146		
No	20	35			21	26		

*Note.* Abbreviations are the same as in Table 1.

**Table 4:** Association analysis of radiotherapy and survival under different expressions of DDX60.

Data	DDX60 expression	Unadjusted (RT vs NRT)		Adjusted (RT vs NRT)	
		HR (95% CI)	*p* values	HR (95% CI)	*p* values
Training	High (*n* = 175)	4.266 (0.943–19.290)	0.060	3.582 (0.627–20.467)	0.054
	Low (*n* = 175)	0.312 (0.123–0.789)	0.014	0.244 (0.064–0.921)	0.040
Validation	High (*n* = 178)	1.848 (0.520–6.571)	0.343	2.421 (0.460–12.773)	0.297
	Low (*n* = 172)	0.314 (0.135–0.729)	0.007	0.199 (0.062–0.646)	0.007
All data	High (*n* = 353)	2.831 (1.074–7.461)	0.035	1.687 (0.485–5.866)	0.411
	Low (*n* = 347)	0.341 (0.186–0.626)	<0.001	0.395 (0.186–0.840)	0.016

*Note.* Adjusted factors: age, history of other malignancies, histologic type, first surgical procedure, TNM stages, ER status, PR status, HER2 status, and chemotherapy.

## Data Availability

The datasets used in the present study are available from The Cancer Genome Atlas database (http://cancergenomec.nih.gov/).

## Supplementary Materials

Supplementary Materials Table S1: comparisons of radiation dose, type, and site in patients under radiotherapy. Figure S1: survival curves under different expression levels of DDX60 for all patients. The total samples were stratified by age (≥60 and <60). The logrank test was employed to estimate *p* values. The number before and after the slash referred to the number of deaths and sample size in subgroups, respectively. Figure S2: survival curves under different expression levels of DDX60 for all patients. The total samples were stratified by T stage (T1/T2 and T3/T4). The logrank test was employed to estimate *p* values. The number before and after the slash referred to the number of deaths and sample size in subgroups, respectively. Figure S3: survival curves under different expression levels of DDX60 for all patients. The total samples were stratified by N stage (N0/N1 and N2/N3). The logrank test was employed to estimate *p* values. The number before and after the slash referred to the number of deaths and sample size in subgroups, respectively. Figure S4: survival curves under different expression levels of DDX60 for all patients. The total samples were stratified by M stage (M0 and M1). The logrank test was employed to estimate *p* values. The number before and after the slash referred to the number of deaths and sample size in subgroups, respectively. Figure S5: survival curves under different expression levels of DDX60 for all patients. The total samples were stratified by chemotherapy (yes and no). The logrank test was employed to estimate *p* values. The number before and after the slash referred to the number of deaths and sample size in subgroups, respectively. Figure S6: the HR values of radiotherapy along with different cutoffs.
