# Supplementary material for: Low Expression of DDX60 Gene Might Associate with the Radiosensitivity for Patients with Breast Cancer
Source: J Oncol. 2020 Jul 20;2020:8309492. doi: 10.1155/2020/8309492 (PMC7387961; doi:10.1155/2020/8309492)
Supplement: Supplementary Materials — Table S1: comparisons of radiation dose, type, and site in patients under radiotherapy. Figure S1: survival curves under different expression levels of DDX60 for all patients. The total samples were stratified by age (≥60 and <60). The logrank test was employed to estimate p values. The number before and after the slash referred to the number of deaths and sample size in subgroups, respectively. Figure S2: survival curves under different expression levels of DDX60 for all patients. The total samples were stratified by T stage (T1/T2 and T3/T4). The logrank test was employed to estimate p values. The number before and after the slash referred to the number of deaths and sample size in subgroups, respectively. Figure S3: survival curves under different expression levels of DDX60 for all patients. The total samples were stratified by N stage (N0/N1 and N2/N3). The logrank test was employed to estimate p values. The number before and after the slash referred to the number of deaths and sample size in subgroups, respectively. Figure S4: survival curves under different expression levels of DDX60 for all patients. The total samples were stratified by M stage (M0 and M1). The logrank test was employed to estimate p values. The number before and after the slash referred to the number of deaths and sample size in subgroups, respectively. Figure S5: survival curves under different expression levels of DDX60 for all patients. The total samples were stratified by chemotherapy (yes and no). The logrank test was employed to estimate p values. The number before and after the slash referred to the number of deaths and sample size in subgroups, respectively. Figure S6: the HR values of radiotherapy along with different cutoffs. [file 8309492.f1.pdf]

Table S1: Comparisons of radiation dose, type and site in patients under radiotherapy.

	Low Expression Group			High Expression Group			Wilcoxon or Fisher exact test P value
	N	Mean(sd)	Median (IQR)	N	Mean(sd)	Median (IQR)	
Dose (Gy)	154	13.0(19.1)	6.0(1.4)	172	10.5(14.7)	6.0(1.3)	0.198
Type							1.000
External	152	-	-	169	-	-	
Other	2	-	-	3	-	-	
Site							1.000
Distant	2	-	-	2	-	-	
Primary	125	-	-	139	-	-	
Regional	27	-	-	31	-	-	

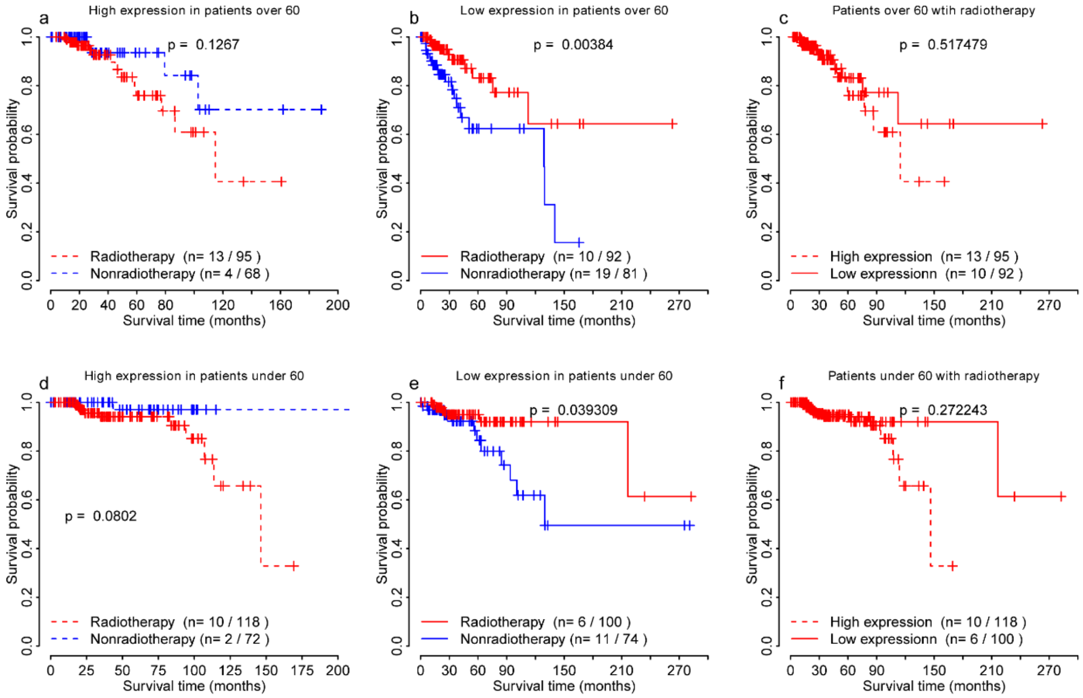

Figure S1: Survival curves under different expression levels of DDX60 for all patients. The total samples were stratified by Age (>=60 and <60). Log-rank test was employed to estimate p values. The number before and after the slash referred to the number of deaths and sample size in subgroups, respectively.

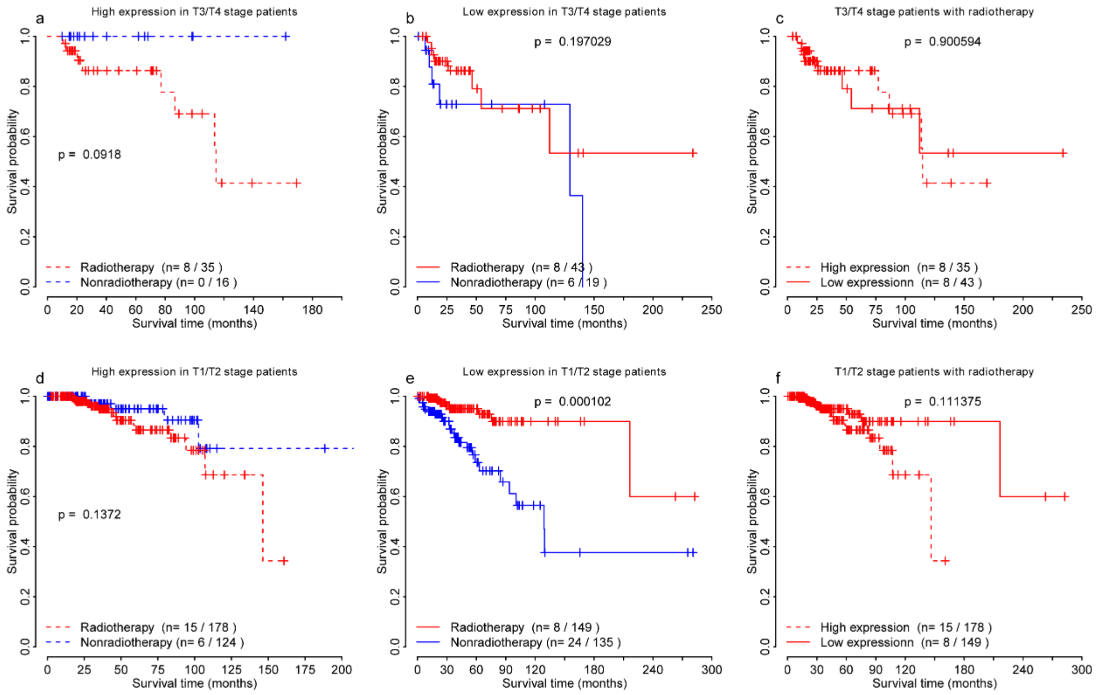

Figure S2: Survival curves under different expression levels of DDX60 for all patients. The total samples were stratified by T stage (T1/T2 and T3/T4). Log-rank test was employed to estimate p values. The number before and after the slash referred to the number of deaths and sample size in subgroups, respectively.

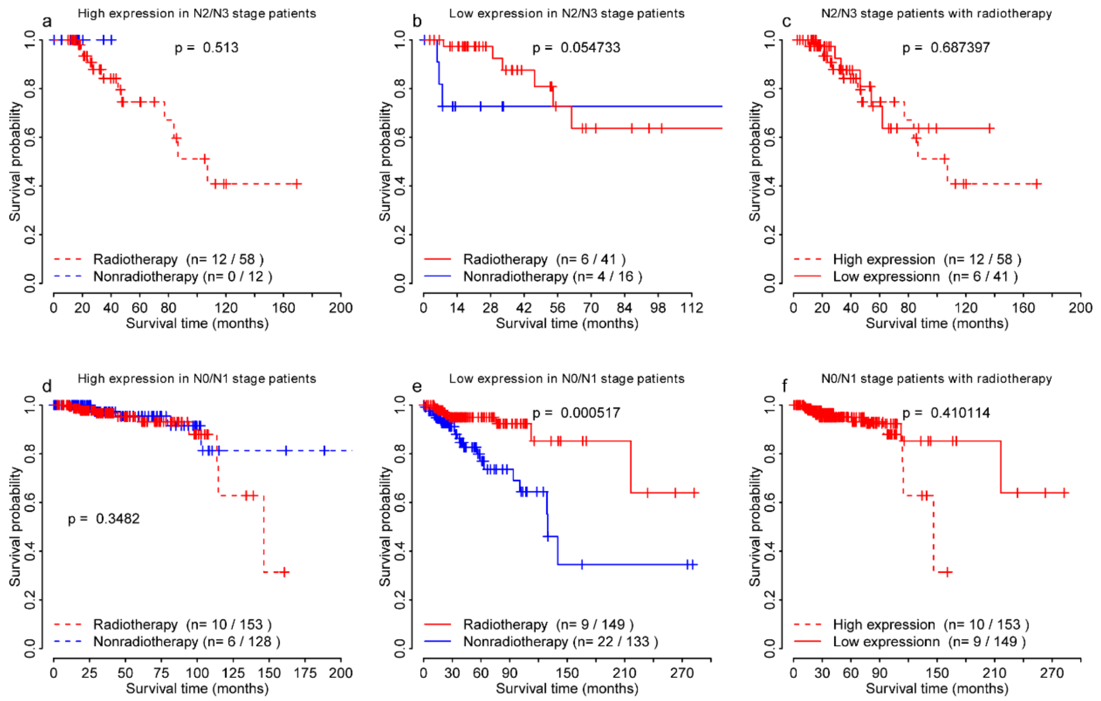

Figure S3: Survival curves under different expression levels of DDX60 for all patients. The total samples were stratified by N stage (N0/N1 and N2/N3). Log-rank test was employed to estimate p values. The number before and after the slash referred to the number of deaths and sample size in subgroups, respectively.

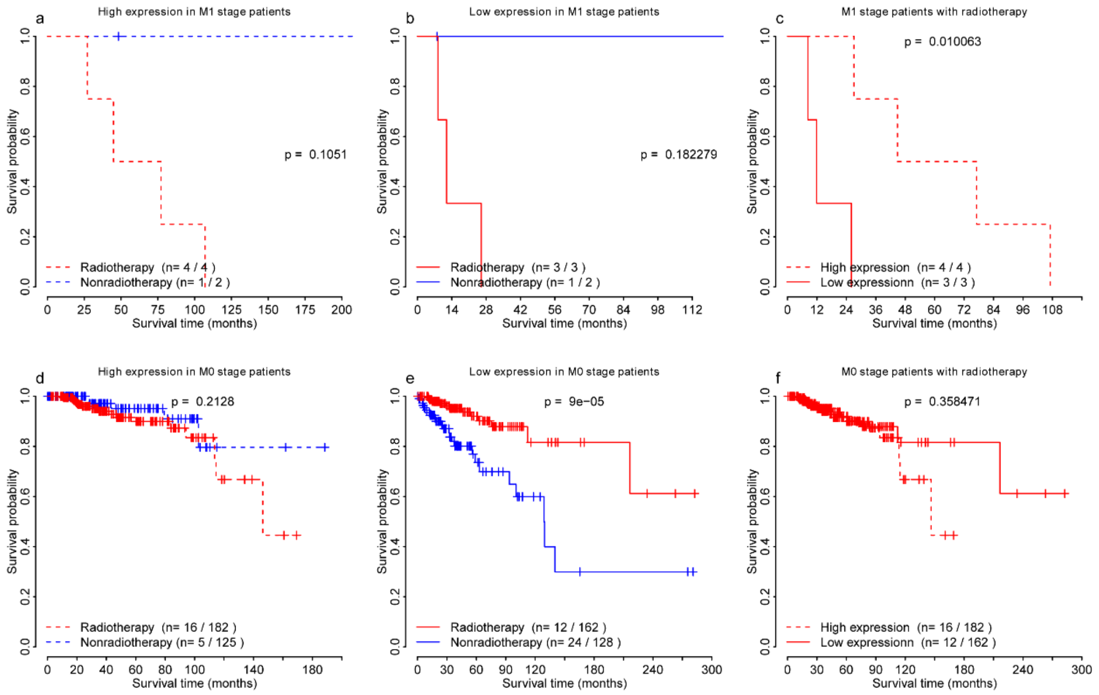

Figure S4: Survival curves under different expression levels of DDX60 for all patients. The total samples were stratified by M stage (M0 and M1). Log-rank test was employed to estimate p values. The number before and after the slash referred to the number of deaths and sample size in subgroups, respectively.

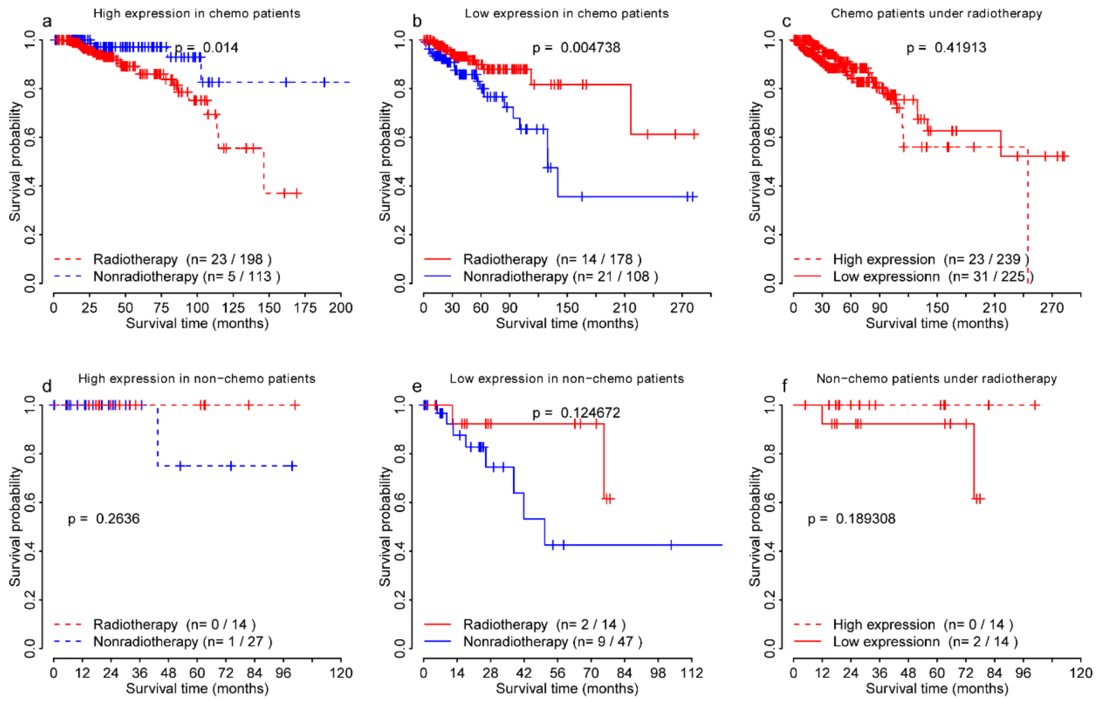

Figure S5: Survival curves under different expression levels of DDX60 for all patients. The total samples were stratified by Chemotherapy (yes and no). Log-rank test was employed to estimate p values. The number before and after the slash referred to the number of deaths and sample size in subgroups, respectively.

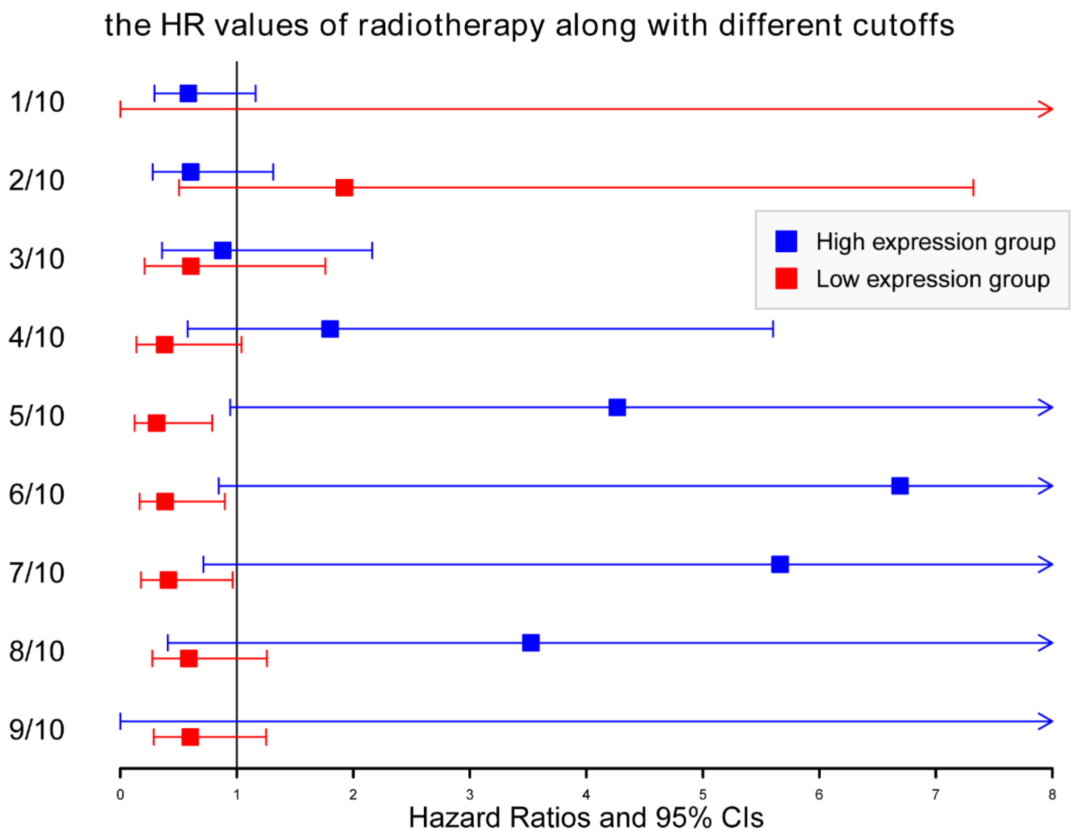

Figure S6: the HR values of radiotherapy along with different cutoffs.
